# Examination of the roles of a conserved motif in the PriA helicase in structure-specific DNA unwinding and processivity

**DOI:** 10.1371/journal.pone.0255409

**Published:** 2021-07-30

**Authors:** Alexander T. Duckworth, Tricia A. Windgassen, James L. Keck

**Affiliations:** 1 Department of Biomolecular Chemistry, University of Wisconsin-Madison, Madison, WI, United States of America; 2 Codexis Inc, Redwood City, CA, United States of America; University of California San Diego, UNITED STATES

## Abstract

DNA replication complexes (replisomes) frequently encounter barriers that can eject them prematurely from the genome. To avoid the lethality of incomplete DNA replication that arises from these events, bacteria have evolved “DNA replication restart” mechanisms to reload replisomes onto abandoned replication forks. The *Escherichia coli* PriA DNA helicase orchestrates this process by recognizing and remodeling replication forks and recruiting additional proteins that help to drive replisome reloading. We have identified a conserved sequence motif within a linker region of PriA that docks into a groove on the exterior of the PriA helicase domain. Alterations to the motif reduce the apparent processivity and attenuate structure-specific helicase activity in PriA, implicating the motif as a potential autoregulatory element in replication fork processing. The study also suggests that multiple PriA molecules may function in tandem to enhance DNA unwinding processivity, highlighting an unexpected similarity between PriA and other DNA helicases.

## Introduction

The essential process of genome duplication is catalyzed by protein complexes called replisomes. During replication, replisome collisions with transcription complexes, DNA damage, or aberrant DNA secondary structures can stall and, in some cases, eject replisomes. Replisome dissociation halts replication and leaves behind abandoned replication forks [1–3]. To rescue prematurely arrested DNA replication processes in these instances, cells have evolved DNA replication restart mechanisms that reload replisomes onto abandoned replication forks.

DNA replication restart in bacteria is facilitated by “primosome” complexes [4, 5]. The PriA DNA helicase is a central component of the primosome and functions to recognize and remodel abandoned replication forks for replisome reloading [6–8]. PriA initiates replication restart by binding to abandoned replication forks in a structure-dependent manner. This allows PriA to act in a sequence-independent fashion, which is important because replication fork abandonment can happen anywhere along a chromosome. PriA structure specificity orients the helicase to unwind lagging strand DNA. Alterations to PriA structural specificity appear to lead to erroneous replication restart events *in vivo* [9, 10], underscoring the importance of proper substrate recognition by PriA. Deleting *priA* altogether severely compromises cells and leads to extreme sensitivity to DNA damage [11].

**Fig 1:**
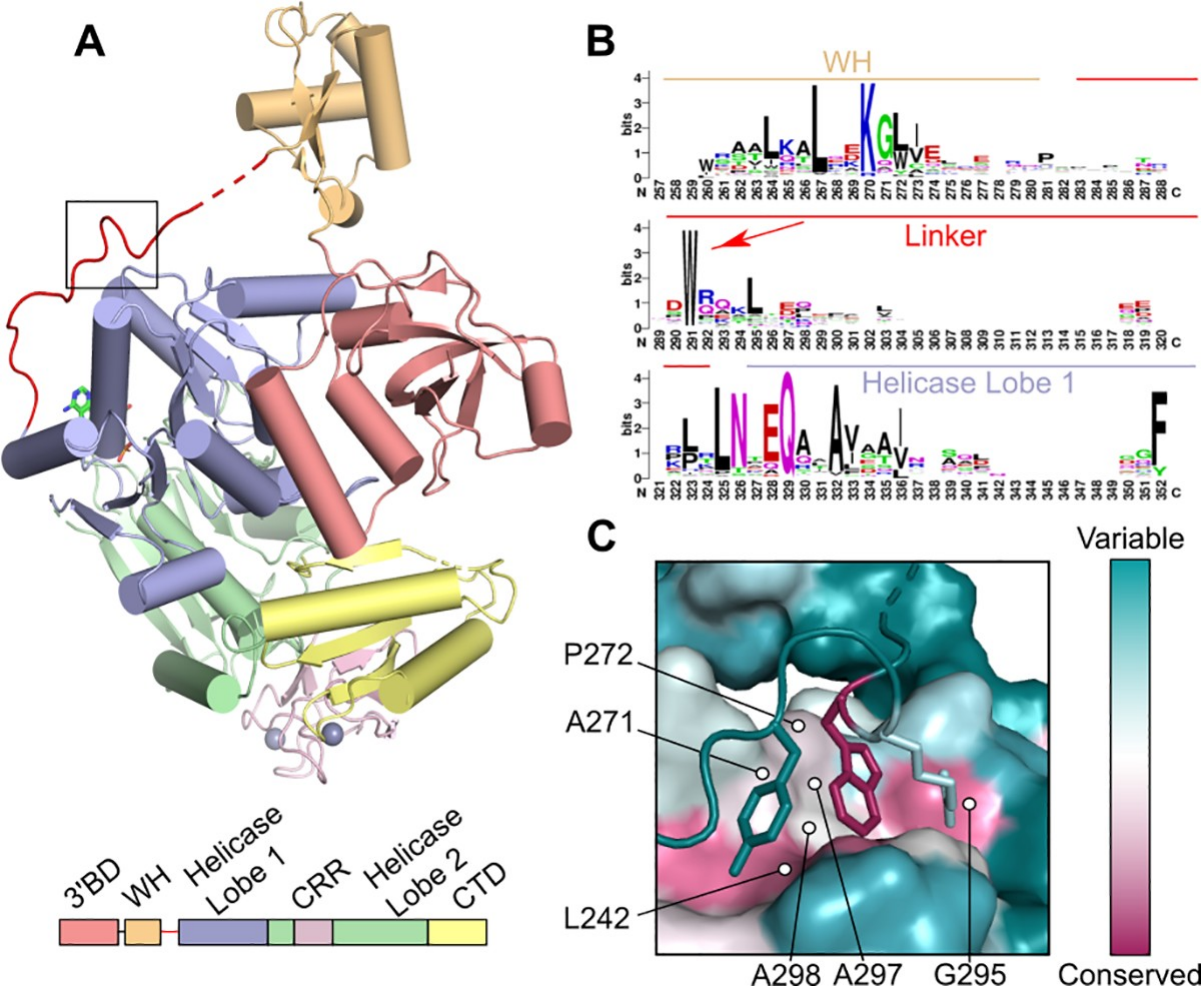
PriA sequence alignment identifies a conserved interaction within PriA. **(A)** Structure of *K*. *pneumoniae* PriA [6, PDB:4NL4] with schematic domain layout. Linker of interest shown in red. Box highlights the linker/HD interface. **(B)** Logo from an alignment of 300 PriA molecules. Parts of the WH and HD are shown along with the linker. Red arrow indicates position of *E*. *coli* Trp186. Note: Sequence alignment numbers do not align with *E*. *coli* numbering due to variations in the lengths of non-conserved regions of PriA proteins from other bacteria. **(C)** Structure of the HD pocket colored by conservation score [20]. Trp186, Arg187, and Tyr190 are shown as sticks and HD pocket residues are labeled. Conservation score color scale is shown.

PriA is comprised of several functional domains (Fig 1) that coordinate to recognize abandoned replication fork structures with high affinity (*K*_D_ ~1-10nM) [6, 9, 12]. The winged-helix (WH) and 3′-binding (3′BD) domains of PriA bind, respectively, to the parental duplex and the 3′-end of the nascent leading strand of replication forks [9, 10]. These contacts are critical for orienting the helicase domain (HD) so that it acts on the lagging strand [9, 13]. Proper relative positioning of the 3′BD and WH domains also appears critical for PriA activity [14]. The presence of single-stranded DNA binding protein (SSB), with which PriA forms a protein complex [6, 15], also helps to orient the PriA HD to the lagging strand [9]. After replication fork recognition, PriA recruits additional proteins to form an active primosome [16–18], which can then direct replisome reloading on the lagging strand template and restart of DNA replication.

While much is known about the roles of individual PriA domains in DNA binding and processing, how PriA biochemical activities are integrated and regulated remains more poorly understood. We have identified a sequence motif that appears to modulate the processivity and structure-specificity of PriA DNA unwinding. This motif (Trp186, Arg187, and Tyr190 of *E*. *coli* PriA) is embedded in a disordered linker that connects the WH and HD (Fig 1). Side chains from these residues dock into a conserved pocket on the HD surface. Sequence changes to linker motif residues produce PriA variants with apparent processivity and structure-specific unwinding defects. These results suggest that the motif serves an autoregulatory role in PriA to enhance the fidelity of abandoned replication fork remodeling for replisome reloading. Interestingly, the results also revealed a phenomenon in which multiple PriA helicases loaded onto a DNA substrate may cooperate to enhance PriA unwinding processivity. This finding highlights the functional similarity between PriA and other helicase families that act in tandem to promote processive unwinding.

## Materials and methods

### Protein expression and purification

N-terminal hexahistidine tagged wild-type *E*. *coli* PriA was purified as described previously [6] with some modifications. His-tagged PriA variants were expressed in BL21(DE3) *E*. *coli* in the presence of 1 mM β-D-1-thiogalactopyranoside for four hours (100 μg/mL ampicillin was included during growth). Cells were resuspended in 40 mL lysis buffer (10 mM HEPES-HCl pH 7.0, 10% glycerol, 500 mM NaCl, 100 mM glucose, 20 mM imidazole, 1 mM β-mercaptoethanol, 1 mM phenylmethylsulfonyl fluoride, 1 mM benzamidine, 1 EDTA-free protease inhibitor tablet), lysed by sonication on ice, and centrifuged for 30 minutes at 30,000 RPM at 4°C.

All purification steps were carried out using an ÄKTA Pure FPLC system (GE) at 4°C. The clarified lysate was loaded directly onto a 5 mL HisTrap FF crude FPLC column (GE) equilibrated in 10 mM HEPES-HCl pH 7.0, 10% glycerol, 500 mM NaCl, 100 mM glucose, 20 mM imidazole, 1 mM β-mercaptoethanol. The column was washed with 50 mL of equilibration buffer and the protein was eluted with 30 mL 10 mM HEPES-HCl pH 7.0, 10% glycerol, 500 mM NaCl, 300 mM imidazole, 1 mM β-mercaptoethanol. Eluate was diluted with 70 mL 10 mM HEPES-HCl pH 7.0, 10% glycerol, 1 mM β-mercaptoethanol to reduce imidazole and NaCl concentrations. Diluted eluate was directly loaded onto a 20 mL HiPrep sulfopropyl sepharose fast flow (SPFF) FPLC column (GE), then eluted using a 100–1000 mM NaCl gradient in elution buffer (10 mM HEPES-HCl pH 7.0, 10% glycerol, 100–1000 mM NaCl, 10 mM dithiothreitol). Fractions enriched for pure PriA were identified using SDS-PAGE, then were pooled, concentrated to 2 mL, and loaded onto a HiPrep S300 FPLC column (GE) equilibrated in 10 mM HEPES-HCl pH 7.0, 10% glycerol, 1000 mM NaCl, 10m M dithiothreitol. Fraction purity was assessed via SDS-PAGE, pure fractions were pooled and concentrated to 2 mL, then dialyzed overnight at 4°C into storage buffer (10 mM HEPES-HCl pH 7.0, 50% glycerol, 500 mM NaCl, 10 mM dithiothreitol) prior to storage.

### Electrophoretic Mobility Shift Assay (EMSA)

EMSAs were performed as described previously [19]. Substrates were prepared by annealing oligonucleotides to make two-stranded (1b-98 and 3L-98, S1 Table), four-stranded (1b-98, 3L-98, b-33, and 11b-38, S1 Table), or extended two-stranded (oTW140 and oTW141) radiolabeled DNA substrates. Briefly, 0.1–10 nM PriA was incubated with 1 nM synthetic replication fork substrate in 50 mM Tris-HCl, pH 8.0, 0.1 mg/mL BSA, 2 mM dithiothreitol, 5 mM EDTA, and 6% glycerol for 30 minutes on ice. Samples were resolved via 4% PAGE before fixing, drying, exposing, and imaging.

### DNA-dependent ATP hydrolysis assays

ATPase assays were performed as described previously [19]. Briefly, PriA or a PriA variant (50 nM) was incubated with 0.1–10,000 nM dT_28_ DNA for 10 minutes at ambient temperature, in 20 mM HEPES-HCl, pH 8.0, 50 mM NaCl, 1 mM β-mercaptoethanol, 5 mM magnesium chloride, 0.1 mg/mL BSA, 2 mM phosphoenolpyruvate, 0.2 mM nicotinamide adenine dinucleotide, 3 U/mL pyruvate kinase, and 4.5 U/mL Lactate Dehydrogenase. ATP (1 mM) was added and A_340 nm_ was monitored for 1 hour at 25°C. Data were analyzed as previously described [19].

### Helicase assays

Helicase assays were performed as described previously [19]. The short and extended two-stranded substrates were prepared as described above for EMSA experiments. Three-stranded substrates were prepared by annealing oligonucleotides 1b-98, 3L-98, and b-33. oTW140, oTW141, and oTW143 were annealed to make the extended three-stranded substrate (S1 Table). Briefly, 0.1–1 nM PriA was incubated with 0.5 nM synthetic replication fork substrate in 50 mM HEPES-HCl, pH 8.0, 0.04 mg/mL BSA, 2 mM dithiothreitol, 2 mM ATP, and 4 mM magnesium acetate for 30 minutes at 37°C. For time course experiments, PriA was 1 nM. Reactions were terminated by adding 20 mM EDTA, 0.5% SDS, 0.2 mg/mL proteinase K, and 2.5 ng/μL cold trap oligonucleotide (3L-98 or oTW141) (final concentrations) and incubating for 30 minutes at 37°C. Samples were resolved by 10% native PAGE, before fixing, drying, exposing, and imaging. Results were quantified using ImageQuant software.

## Results

### A conserved linker motif docks into a hydrophobic groove in the PriA helicase domain

PriA is comprised of five major domains that confer the ability to recognize and remodel abandoned replication forks with high specificity [6, 9] (Fig 1A). However, elements within PriA that help to regulate its functions have not been well defined. To identify potential regulatory elements, 300 bacterial PriA sequences were aligned to find evolutionarily conserved segments that lacked known functions. The alignment revealed a well conserved Trp residue (Trp186 in *E*. *coli* PriA) within a linker that connects the WH and HD [20, 21] (Fig 1B). Examination of the crystal structures of *Klebsiella pneumoniae* and *E*. *coli* PriA [6, 9] showed that Trp186 and two less well conserved residues (Arg187 and Tyr190 in *E*. *coli* PriA) form a motif that docks into a conserved pocket on the PriA HD (Fig 1C). The side-chain nitrogen atoms of Trp186 and Arg187 are within hydrogen-bonding distance of the carbonyl oxygen atom of Gly295 from the pocket (3.2Å and 3.1Å, respectively) and the hydrophobic regions of Trp186 and Tyr190 are accommodated by hydrophobic surfaces from the HD (Fig 1C). The pocket is ~25 Å from the HD ATPase active site, which raises the possibility that docking is important for PriA biochemical functions. The position of the binding pocket in the HD and the proximity of the linker motif to the 3′BD and WH domains led us to hypothesize that the linker/HD interaction may be important for regulating PriA activity.

### Linker variants retain wild-type DNA binding abilities but have modestly reduced DNA-dependent ATPase activity

To test the hypothesis that linker motif docking into the HD plays a role in PriA activity, single-site variants of *E*. *coli* PriA (Trp186Ala, Arg187Ala, or Tyr190Ala) and a triple variant where all three residues were changed to Ala were constructed, purified, and tested in a panel of biochemical assays. The variants were designed to destabilize linker docking onto the HD, with the triple variant having the most impactful change.

**Fig 2:**
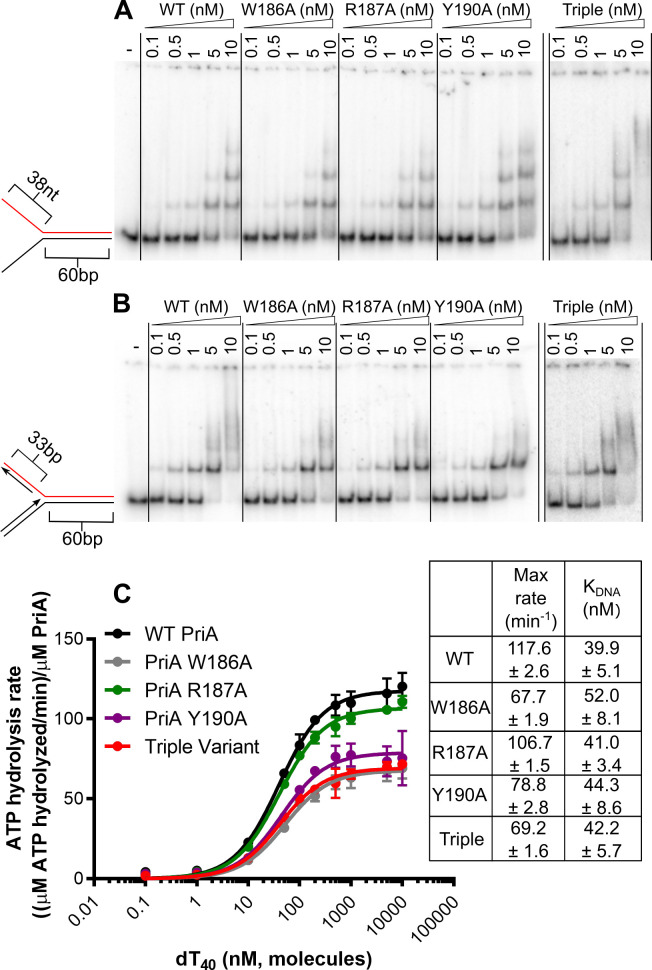
Linker variants retain fork binding but hydrolyze ATP less efficiently. **(A and B)** EMSAs with PriA variants at indicated concentration on a radiolabeled two-stranded fork (1 nM, molecules) **(A)** or four-stranded fork (1 nM, molecules) **(B)**. Substrates are depicted on left with radiolabeled strand shown in red. **(C)** ATP hydrolysis rate as a function of [dT_40_] for each PriA variant (50 nM, molecules). Mean max rate and K_DNA_ values derived from three replicates are listed, with error representing SD. Gels are representative of at least three replicates.

The ability of each of the PriA variants to bind DNA was tested using an electrophoretic mobility shift assay (EMSA). Wild-type *E*. *coli* PriA or variants were titrated with either a two-stranded synthetic replication fork structure containing 60-bp parental double-stranded DNA (dsDNA) and 38-nt single-stranded DNA (ssDNA) template strands or a four-stranded fork containing 60-bp parental duplex and 38- and 33-bp nascent leading and lagging strands, respectively [22] (Fig 2A and 2B). The 5-nt lagging strand gap was included in the four-stranded fork since PriA requires a ~5nt ssDNA gap between the nascent lagging strand and fork junction for full unwinding activity [23]. As the concentrations of wild-type or variants PriA were increased, the two-stranded fork was observed to shift to three lower-mobility bands (Fig 2A). This EMSA pattern likely reflects PriA binding to the fork junction and each ssDNA arm at high concentrations. The concentration dependence of the EMSA pattern is unchanged among all three single variants as well as the triple variant, suggesting that the sequence changes do not impact DNA binding affinity.

On the four-stranded fork, one dominant shifted species is observed, suggesting that PriA and the variants can only stably bind to the fork junction of this DNA structure (Fig 2B). A smear is observed at higher concentrations which may represent weaker or more transient binding to dsDNA regions in the substrate. Similar EMSA behavior has been noted previously for wild-type *E*. *coli* PriA [9]. As with the two-stranded fork, the single and triple variants all show similar concentration-dependent DNA binding, suggesting alteration of the linker sequence does not significantly affect binding to abandoned replication forks.

To test whether the linker residues are important for catalytic activity in PriA, we measured DNA-dependent ATPase rates for wild-type PriA and the linker variants. The PriA proteins were incubated with increasing concentrations of dT_40_ ssDNA and ATPase rates were measured (Fig 2C). Although no significant difference in the concentration of ssDNA needed to promote half maximal ATPase rates (K_DNA_) was observed among the variants tested, the maximum ATPase rates of the Trp186Ala and Tyr190Ala variants were reduced by 1.7- and 1.5-fold, respectively. These modest decreases are not compounded in the triple variant which also showed a 1.7-fold reduction. Overall, these results suggest that Trp186 and Tyr190 are needed for maximal ATPase and ssDNA-dependent ATPase rates but are dispensable for DNA binding.

### PriA linker variants are defective in unwinding the parental duplex of a synthetic fork

**Fig 3:**
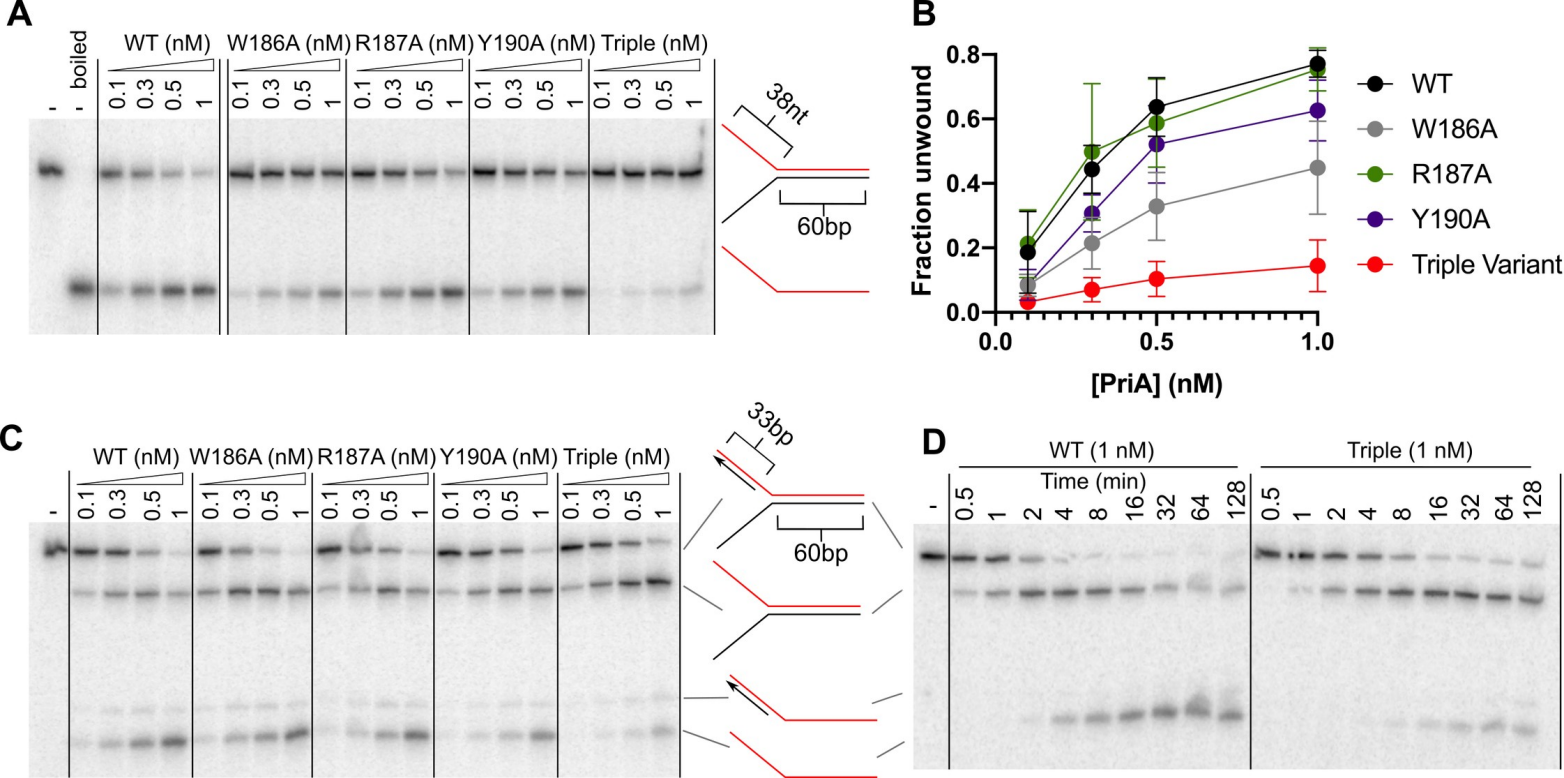
Parental duplex unwinding is impaired in PriA linker variants. **(A)** Unwinding products from incubation of two-stranded fork (0.5 nM, molecules) with PriA variants at concentrations shown and 2mM ATP. **(B)** Quantification of unwinding using ImageQuant software. Data points depict the mean of 4 replicate experiments, with error bars representing SD. **(C and D)** Unwinding products from incubation of three-stranded fork (0.5 nM, molecules) with PriA variants at concentrations shown and 2mM ATP. Assays are shown as 30-minute end-point **(C)** or time-course **(D)** experiments. Substrates are depicted with radiolabeled strand shown in red. Gels are representative of at least three replicates.

To determine whether the PriA linker motif residues are important for DNA replication fork unwinding, the activities of the PriA variants were assessed in helicase assays that measure strand-specific unwinding. Helicase activities were measured on two different substrates: the two-stranded fork used in the prior DNA binding experiments and a three-stranded fork in which a third 33-nt oligonucleotide was annealed to the 5′ ssDNA extension of the two-stranded fork to create a duplex lagging strand (Fig 3). As observed previously [9], the two-stranded fork is robustly unwound by wild-type PriA in a concentration-dependent manner (Fig 3A). Among the PriA variants, the Arg187Ala and Tyr190Ala proteins unwound the substrate as efficiently as wild-type PriA. In contrast, the Trp186Ala, variant had modestly reduced helicase activity and the triple variant PriA displayed significantly reduced DNA unwinding activity (Fig 3A and 3B).

The three-stranded fork contains two duplex regions that can be unwound by PriA, which provides a test of whether PriA variants retain the ability to preferentially unwind the nascent lagging strand [9]. Three unwinding products are apparent with the three-stranded fork (Fig 3C and 3D): (*1*) unwinding the nascent lagging strand alone produces the two-stranded fork, (*2*) unwinding the parental duplex but not the nascent lagging strand produces a structure containing regions of both ssDNA and dsDNA, and (*3*) unwinding the nascent lagging strand and the parental duplex produces ssDNA (Fig 3C and 3D). During the 30-minute reaction, wild-type PriA unwinds the DNA to form each of the products in a concentration-dependent manner, with the completely unwound ssDNA product as the dominant product at the highest PriA concentration tested (Fig 3C). While each of the single-site variants unwound the three-stranded fork similarly to wild-type PriA, the triple variant primarily produced the two-stranded fork as the major product. This demonstrated that the triple variant retained helicase activity but had very little activity on the two-stranded fork substrate.

A time-resolved helicase assay was used to further probe DNA unwinding by wild-type PriA and the triple variant (Fig 3D). As has been observed previously with the three-stranded substrate [9], wild-type PriA preferentially unwinds the nascent lagging strand first to produce a two-stranded fork intermediate. The two-stranded fork is then further unwound to form the ssDNA product. In comparison, the PriA triple variant retains the ability to unwind the lagging strand (albeit at a slightly slower overall rate) but does not efficiently unwind the two-stranded fork product. This is evidenced by the large fraction of two-stranded fork product remaining after two hours of incubation. These data indicate that the PriA linker sequence is important for unwinding of parental duplexes in the substrates tested.

### The triple linker variant can unwind parental duplex if the ssDNA portion of the fork is extended

The PriA variant unwinding results are consistent with at least two possible defects with the triple variant. First, the variant may orient the HD differently on fork substrates leading to a difference in its DNA-structure specific activities. Second, since the variant can unwind a 33-bp substrate (lagging strand duplex) but is less efficient with a 60-bp substrate (parental duplex), it may have a processivity defect. We note that the experiments testing unwinding were not performed under single turnover conditions, which precluded measurement of definitive processivity values. As such, the term apparent processivity is used below to discuss differences that were observed.

**Fig 4:**
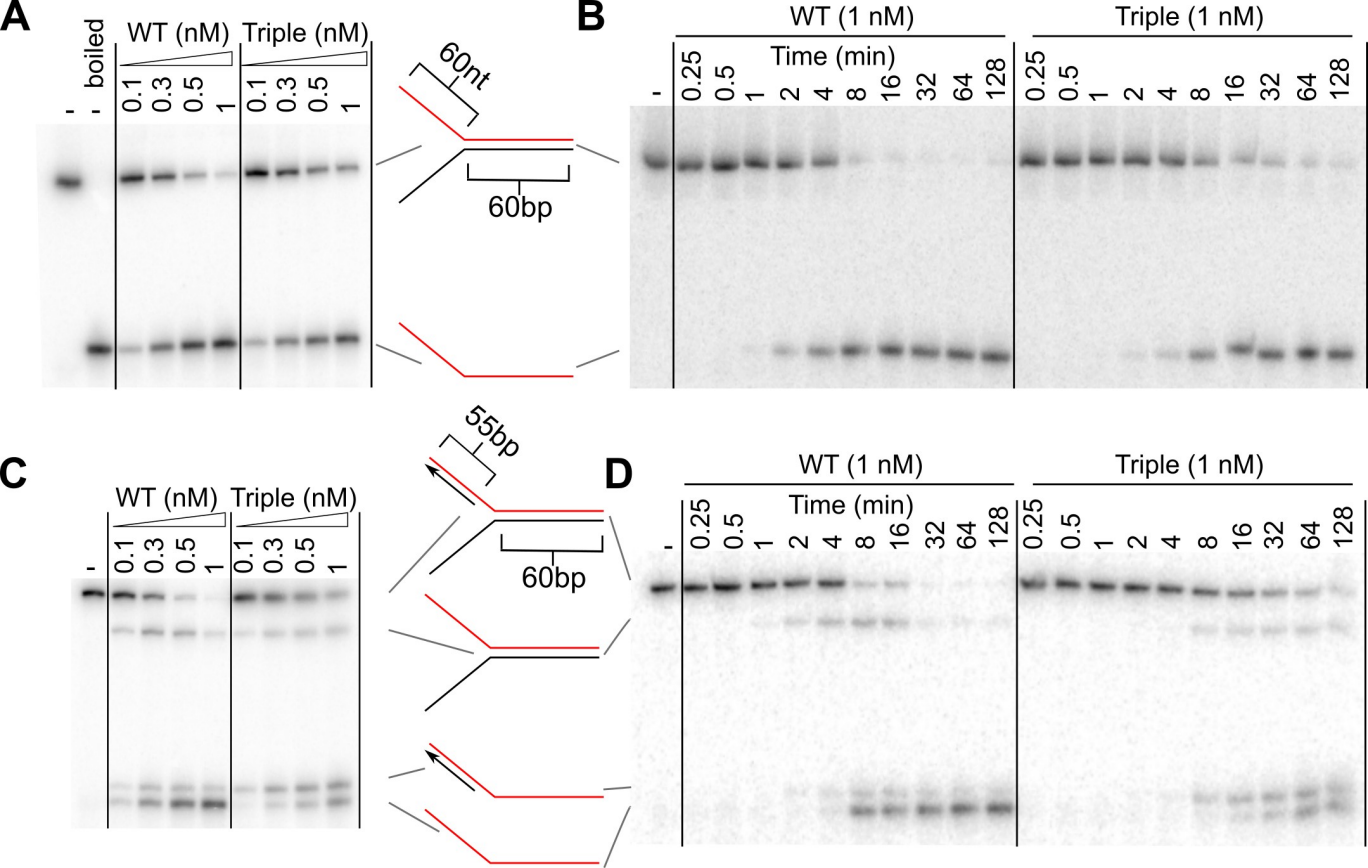
Triple linker variant can unwind parental duplex of extended forks. **(A and B)** Unwinding products from incubation of extended two-stranded fork (0.5 nM, molecules) with PriA variants at concentrations shown and 2mM ATP. Assays are shown as 30-minute end points **(A)** or time-course **(B)** experiments**. (C and D)** Unwinding products from incubation of extended three-stranded fork (0.5 nM, molecules) with PriA variants at concentrations shown and 2mM ATP. Assays are shown as 30-minute end points **(C)** or time-course **(D)** experiments. Substrates are depicted with radiolabeled strand shown in red. Gels are representative of at least three replicates.

To distinguish between these possibilities, we constructed extended two- and three-stranded forks with 60-bp leading, lagging, and parental DNA arms and assayed unwinding by wild-type PriA and the triple variant (Fig 4). We reasoned that if the variant is defective in structure-specific fork processing, the assay results would appear similar to the experiment with shorter substrates in Fig 3. However, if the variant has an apparent processivity defect, we predicted that it would not efficiently unwind the fork due to the **increased length of the extended substrates.**

In contrast to both hypotheses, the PriA triple variant unwound the longer two-stranded fork nearly as well as wild-type PriA, although the variant unwound at a slightly slower rate (Fig 4A, 4B, and S1 Fig). This was evident in both concentration-dependent and time-resolved helicase assays. On the extended three-stranded fork containing a 55-bp duplex lagging strand, wild-type PriA unwound with similar efficiencies to those observed with the shorter three-stranded fork leaving mostly ssDNA after 30 mins (Fig 4C). The triple variant was also able to fully unwind this substrate, leaving ssDNA as well as a significant amount of product containing regions of ssDNA and dsDNA. In a time-course assay with the extended three-stranded substrate, wild-type PriA unwinds the nascent lagging strand preferentially and subsequently unwinds the two-stranded fork. In contrast, the triple variant shows no preference and unwinds the parental and lagging strand duplexes at slightly slower rates than wild-type PriA (Fig 4D). These results indicate that the triple variant can unwind a longer (60-bp) parental duplex, but it requires long (>38-nt) ssDNA arms to do so. Also, the triple variant appears to have lost specificity for unwinding the lagging strand of a DNA replication fork. Interestingly, the loss of a preference for the lagging strand in the triple variant mirrors the defect observed in a variant in which the WH domain has been removed from PriA [9].

### PriA triple variant unwinding of a 60-bp substrate correlates with loading of additional PriA molecules

**Fig 5:**
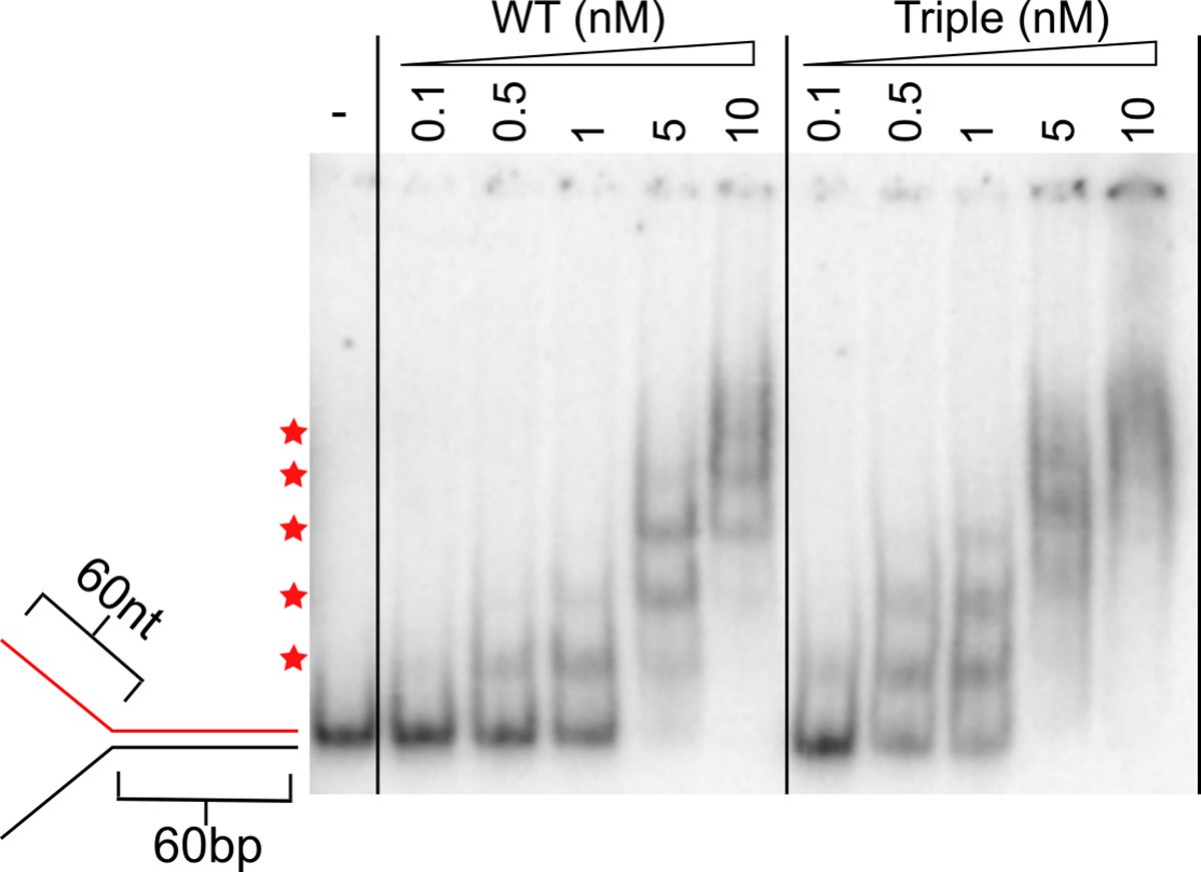
Extended fork substrates allow loading of additional PriA molecules. EMSA with PriA variants at indicated concentrations on extended two-stranded fork (1 nM, molecules). Substrate is depicted with radiolabeled strand shown in red. Red stars indicate locations of distinct, shifted bands. Gel is representative of at least three replicates.

Eoff and Raney previously described a phenomenon in which multiple helicase molecules operating on a common DNA substrate can increase the apparent processivity of unwinding of Dda helicase [24]. In this model, when the actively unwinding helicase at the ssDNA/dsDNA junction dissociates, the next molecule on the DNA rapidly translocates to the junction and continues the unwinding reaction. Such a model for PriA could explain the ability of the triple variant to efficiently unwind the extended forks but not their shorter counterparts. Moreover, this model predicts that more PriA molecules should be able to bind the extended DNA substrates relative to shorter substrates. To test this prediction, we performed an EMSA with the extended two-stranded fork (Fig 5). On this substrate, five shifted bands are present in the higher concentration lanes. This contrasts with the three shifted bands observed with the shorter two-stranded substrate (Fig 2A). Since the only difference between these two substrates is the length of the ssDNA segments, these results indicate that more PriA molecules can bind the ssDNA regions of the longer fork than the shorter one. This observation is consistent with the notion that multiple PriA molecules could cooperate to enhance the apparent processivity of DNA unwinding.

## Discussion

We have investigated the roles of a motif comprised of Trp186, Arg187, and Tyr190 in regulating the biochemical activities of the *E*. *coli* PriA helicase. The side chains of these residues, which are in a linker connecting the WH and HD of PriA, dock into a conserved pocket on the surface of the HD (Fig 1). Biochemical changes associated with alterations in the linker/HD interaction were measured in three single-site Ala linker variants and a triple variant where all three residues were changed to Ala. While all four variants exhibited wild-type levels of DNA binding, the triple variant displayed the most pronounced reduction in DNA-dependent ATPase and DNA unwinding activities. The defects included impaired DNA unwinding structure specificity and apparent processivity. Interestingly, lengthening of the ssDNA adjacent to duplex DNA allowed loading of multiple PriA proteins and led to an enhancement in the overall apparent processivity of the PriA triple variant. These results suggest an important role for linker motif/HD interactions in regulating PriA activity and indicate that multiple PriA molecules can function together on a given substrate to enhance DNA unwinding processivity.

With respect to processivity, analysis of helicase activity in the PriA triple variant uncovered an apparent defect in unwinding long duplex DNA that is dependent upon the length of adjacent ssDNA segments. The variant was not able to efficiently unwind a 60-bp duplex when the ssDNA arms were 38-nt, but unwinding was restored when the arms were extended to 60-nt (Figs 3 and 4). EMSA analysis showed that more PriA molecules could simultaneously bind to the longer two-stranded fork than the shorter fork (Fig 5). This increased binding correlated to increased unwinding activity in the triple variant.

**Fig 6:**
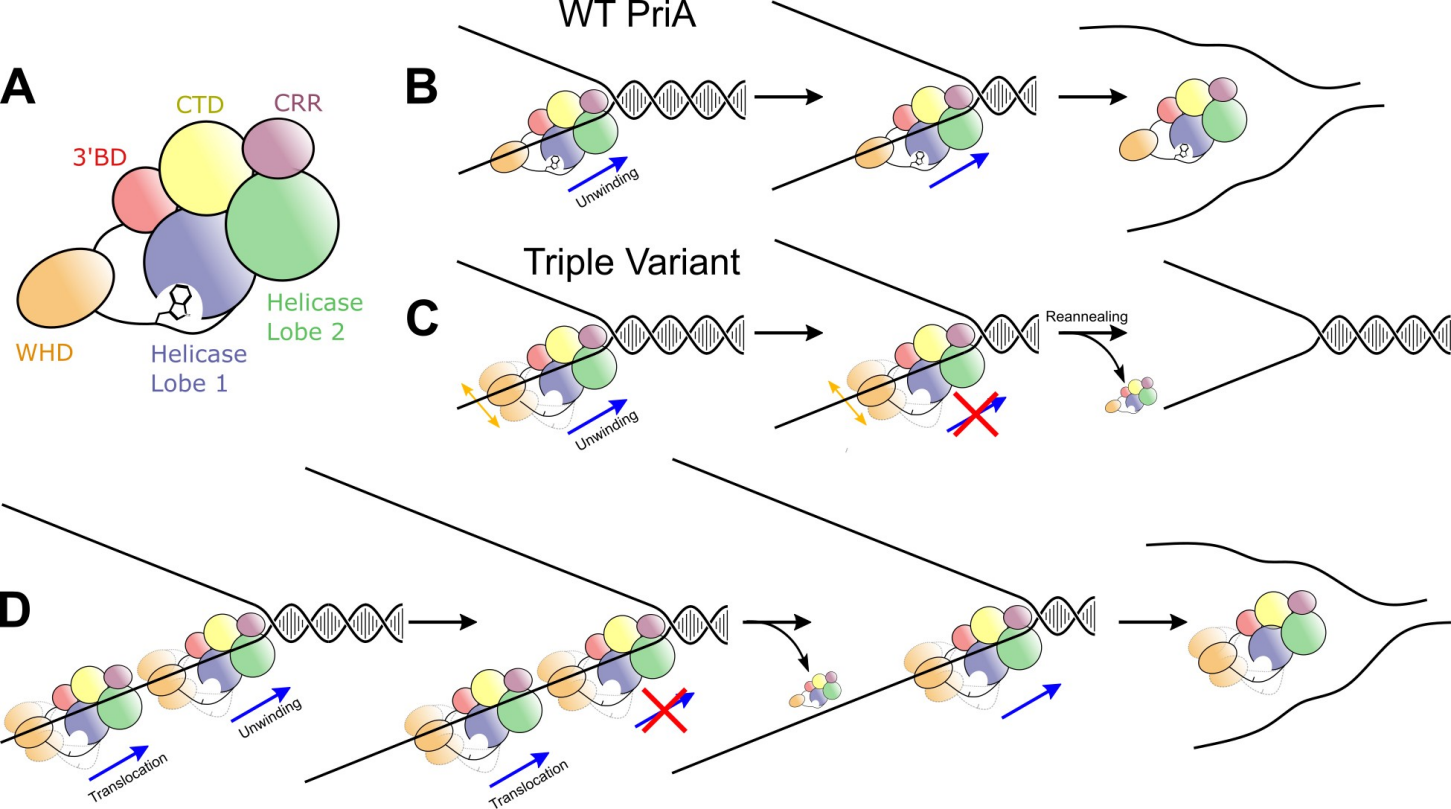
PriA linker variants may be less processive, requiring multiple molecules to unwind forks. **(A)** Cartoon of PriA domain layout colored as in Fig 1A. **(B)** Model depicting one molecule of wild-type PriA unwinding long tracts of dsDNA. **(C)** PriA linker variants are less processive than wild-type PriA. One molecule is insufficient to unwind long tracts of DNA. **(D)** Multiple aligned linker variant PriA molecules can unwind long dsDNA tracts. If one molecule partially unwinds the duplex but falls off, other molecules can replace it.

The PriA unwinding and DNA binding behavior described suggests that it may operate by similar mechanisms to that proposed by Eoff and Raney for the Dda helicase [24]. For Dda, the helicase displayed different unwinding activities in a manner that depended on the length of the ssDNA that was adjacent to a duplex. A longer substrate that can accommodate multiple helicases was unwound with a higher apparent processivity than a shorter substrate with fewer bound helicase molecules. Our PriA data parallel that of Dda. Fig 2B shows the four-stranded fork, which lacks significant ssDNA for PriA loading, stably forms a single shifted PriA/DNA species. Exposure of ssDNA in the template arms in the short two-stranded fork leads to two new shifted species (Fig 2A), with additional PriA molecules likely binding to each ssDNA arm. This two-stranded substrate is fully unwound by wild-type PriA but is not efficiently unwound by the triple variant. The two-stranded fork with extended ssDNA template arms forms even more PriA/DNA species (Fig 5), suggesting that additional molecules of PriA can bind each ssDNA region. The increased number of PriA molecules binding the ssDNA regions correlated with an increased ability of the triple variant to unwind the 60-bp duplex region. Whereas wild-type PriA molecules can efficiently unwind the 60-bp dsDNA when a short ssDNA loading strand is present, the triple linker variant cannot (Fig 6B and 6C). Our data are consistent with a model in which the triple linker variant requires multiple helicase molecules bound to the 3′ ssDNA to efficiently unwind the 60-bp region (Fig 6D). Previous publications have shown that PriA helicase efficacy decreases as the length or GC-content of a dsDNA substrate increases [14, 25]. The results described above suggest that multiple PriA molecules can cooperate to unwind difficult substrates.

Although Trp186Ala and Tyr190Ala each had lower ATPase activity, this reduction was not compounded in the triple variant, which had a similar ATPase activity to Trp186Ala. However, the triple variant did display a compounded change in helicase activity relative to the single variants. We hypothesize that this discrepancy is due to the different activities required in these two assays. In ATPase assays, PriA molecules are simply translocating on a ssDNA substrate whereas in helicase assays the molecules are required to mechanically separate two DNA strands. Thus, the triple variant may not convert the chemical energy of ATP hydrolysis as efficiently into unwinding energy as the single variants.

The PriA triple variant also displayed a defect in structure-specific DNA unwinding relative to the wild-type protein. As has been observed previously [9], wild-type PriA selectively unwound the lagging strand before unwinding the parental duplex at a replication fork structure (Fig 4D). However, the triple variant PriA was not selective. Instead, it unwound the nascent lagging strand and parental duplex without preference (Fig 4D). As mentioned above, this result mirrors the effect of deleting the WH domain from PriA [9]. The PriA WH domain binds to the parental duplex and appears to serve as an important substrate specificity domain by directing the PriA HD to the nascent lagging strand [9]. This arrangement helps to facilitate preferential unwinding of the lagging strand as observed in Figs 3D and 4D. When the WH is deleted, the HD of the resulting variant is not targeted selectively to the nascent lagging strand and instead shows an unwinding profile that is strikingly similar to that of the triple variant in Fig 4D [9]. These results suggest docking of the linker motif in the PriA HD is important for orientation functions of the WH. Undocking of the linker may enhance dynamics relative to the rest of PriA, which could limit its ability to constrain positioning of the HD.

## Supporting information

S1 Fig Quantification of Fig 4A. (TIFF)

S1 Raw images Uncropped gel images for Figs 2–5. For gels in which only a portion was used in the respective figure, the portions that were used are boxed. Gel lanes not included in the final figures are marked with a red "X". All images were obtained using a phosphorimager. (A and B) Fig 2A. (C and D) Fig 2B. (E and F) Fig 3A. (G) Fig 3C. (H) Fig 3D. (I) Fig 4A and 4C. (J) Fig 4B. (K) Fig 4D. (L) Fig 5.
(PDF)

S1 Table Oligonucleotides used to prepare synthetic forks. (PDF)
