# Supplementary figures and images for: Examination of the roles of a conserved motif in the PriA helicase in structure-specific DNA unwinding and processivity
Source: PLoS One. 2021 Jul 30;16(7):e0255409. doi: 10.1371/journal.pone.0255409 (PMC8323898; doi:10.1371/journal.pone.0255409)

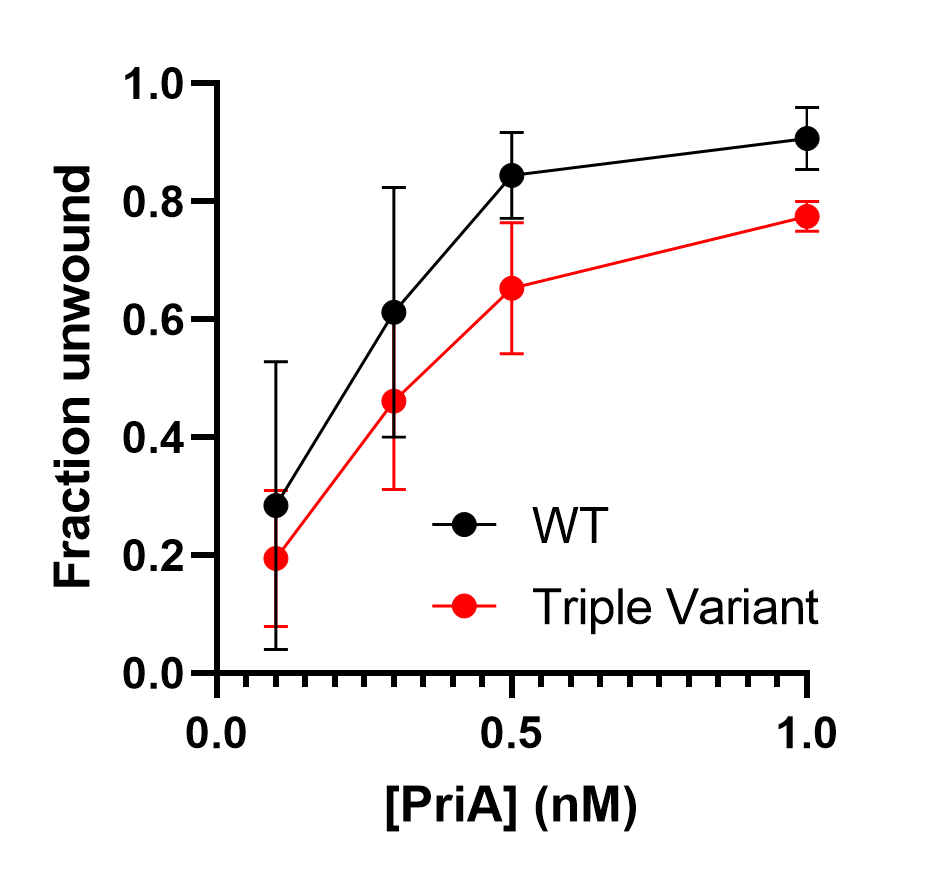

Supplement: S1 Fig — (TIFF) [file pone.0255409.s001.tiff]

Figure S2

A.

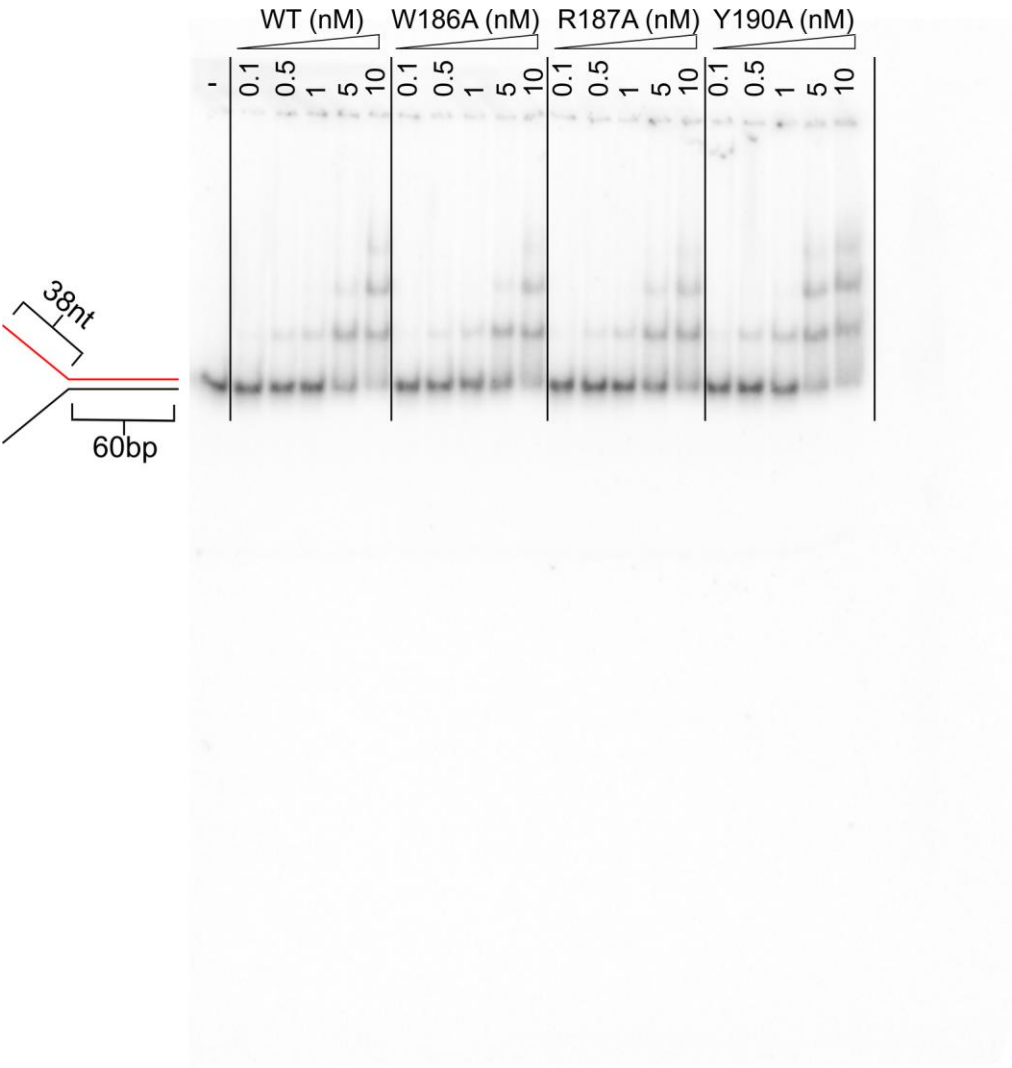

**B.**

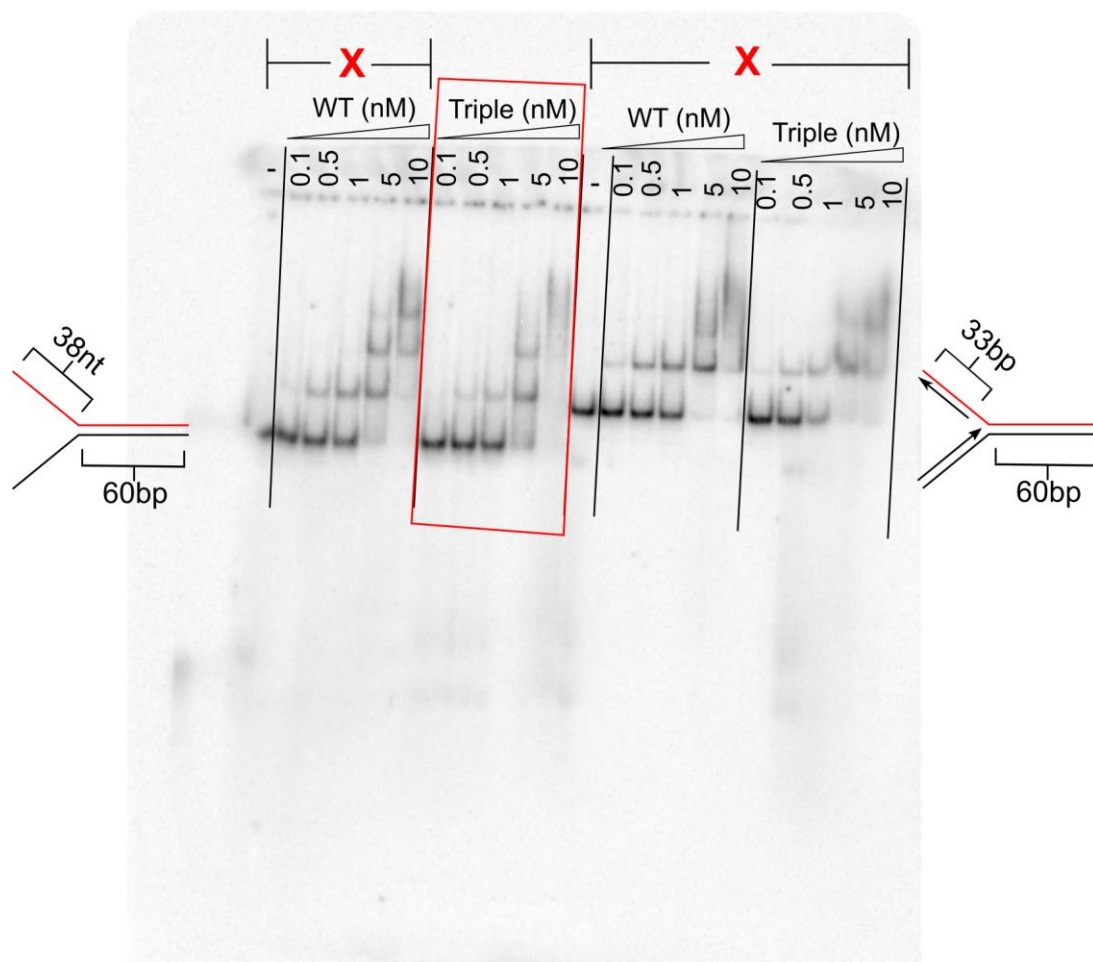

C.

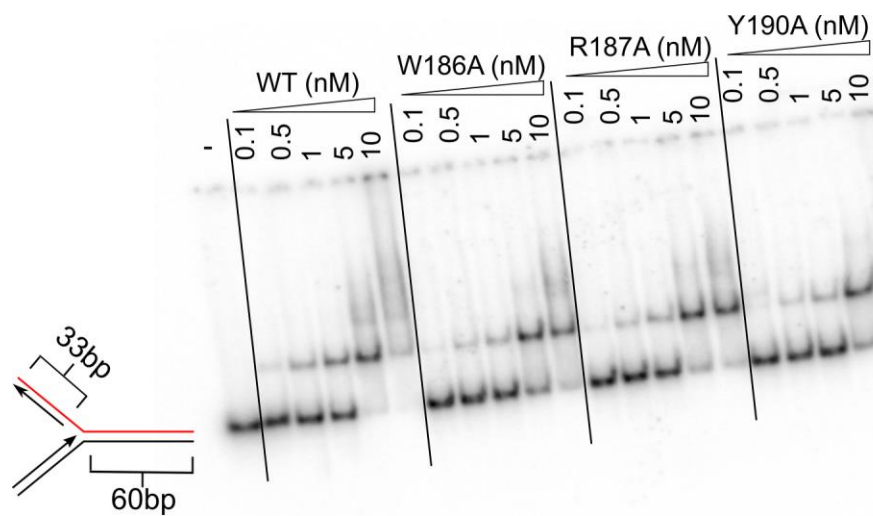

D.

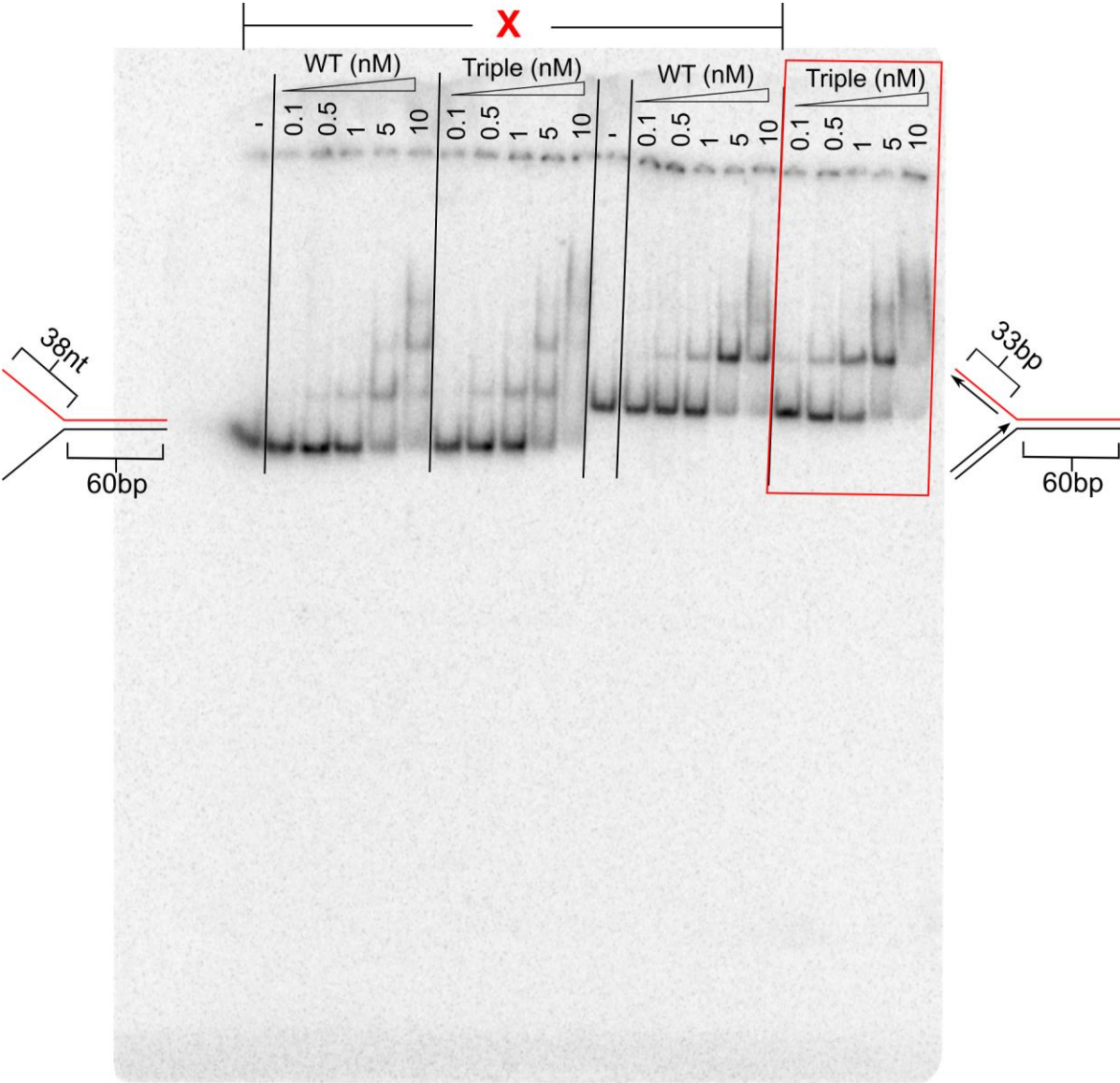

E.

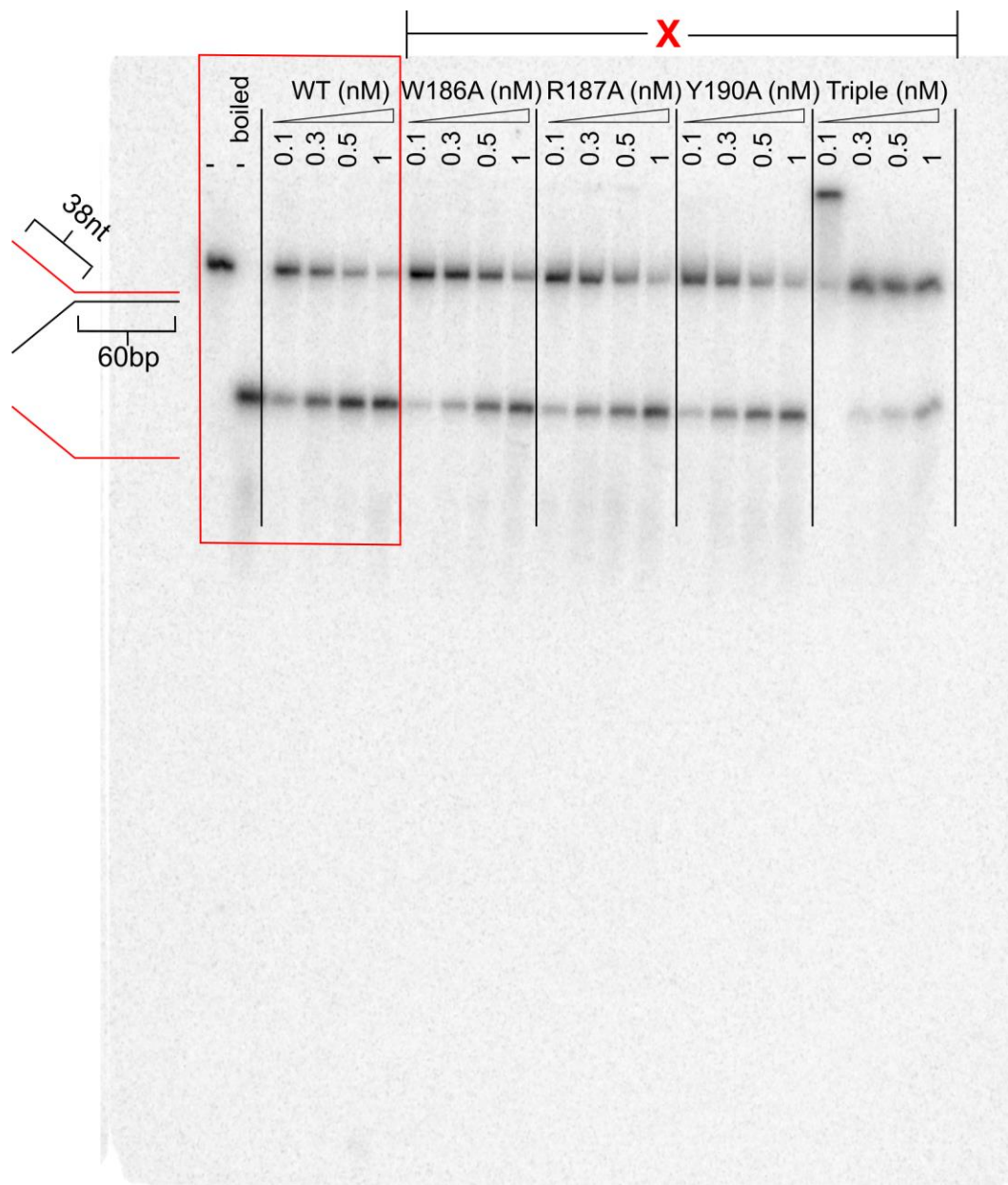

F.

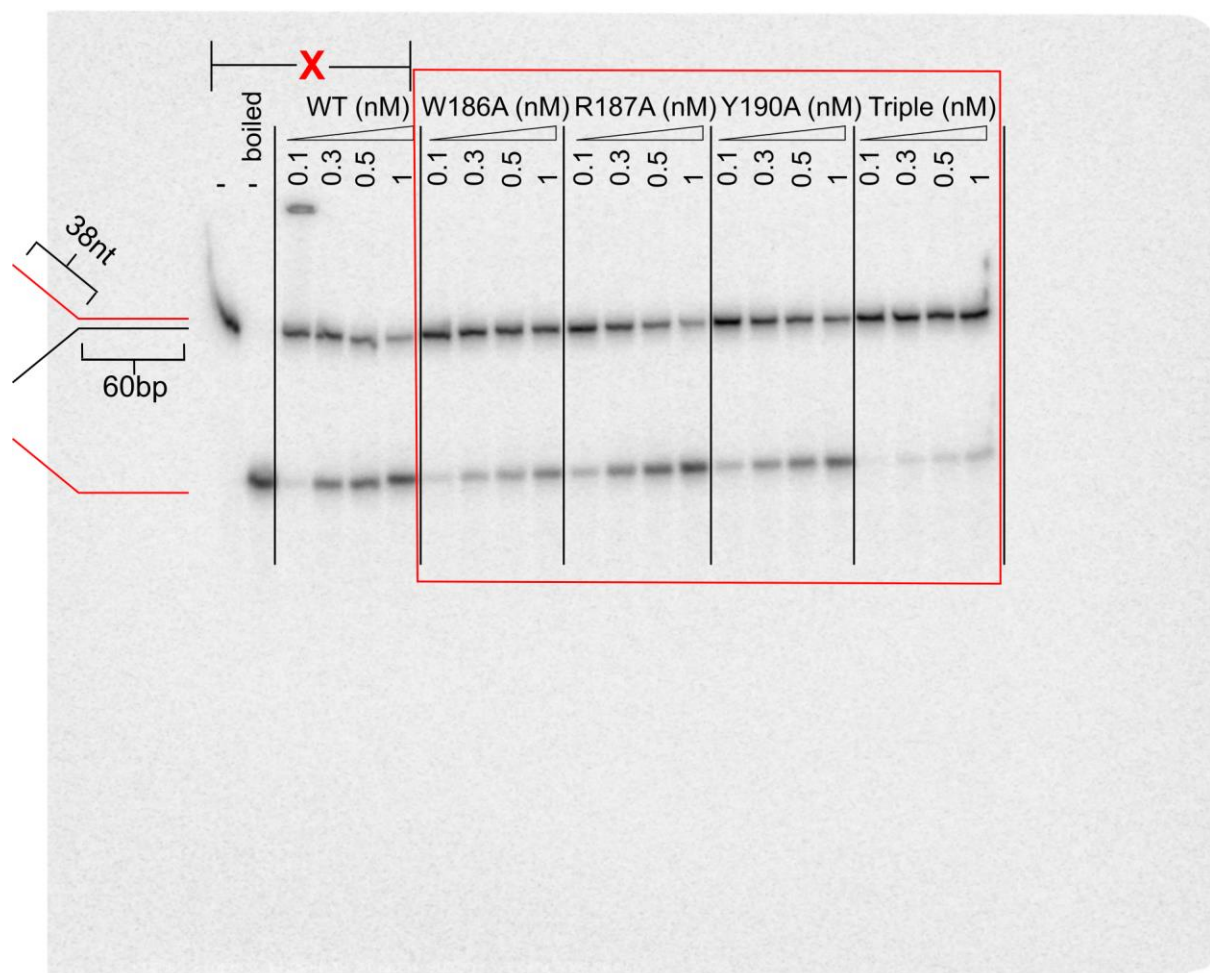

**G.**

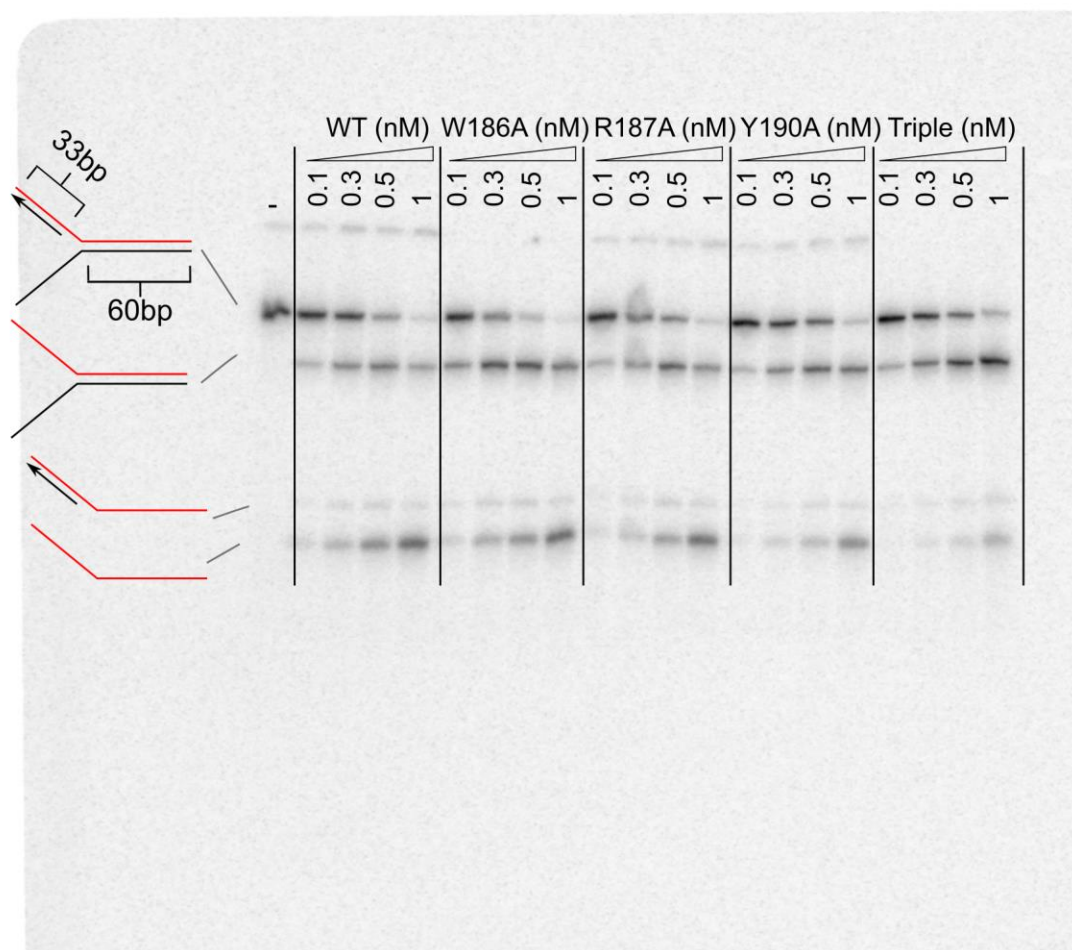

H.

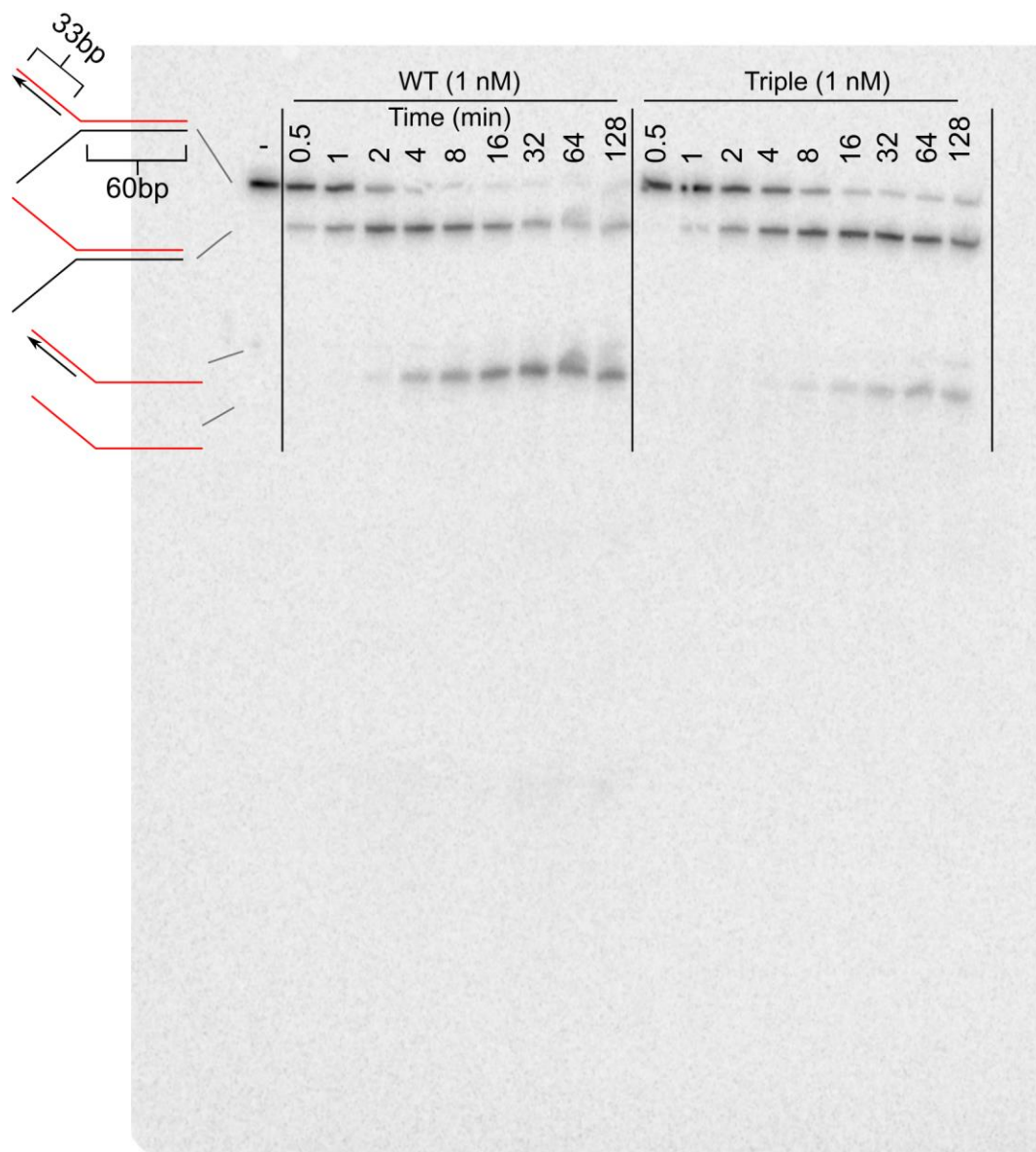

I.

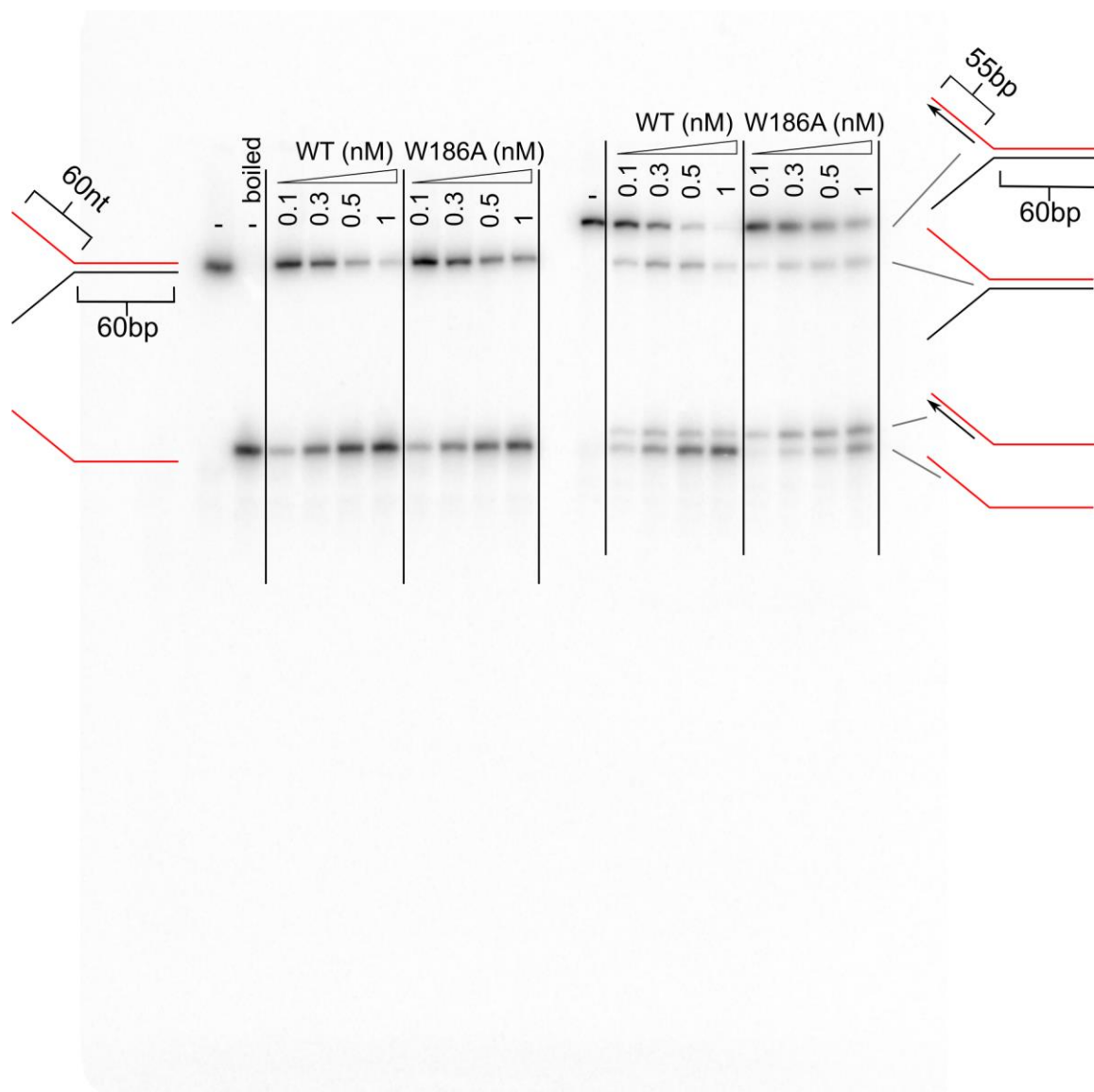

J.

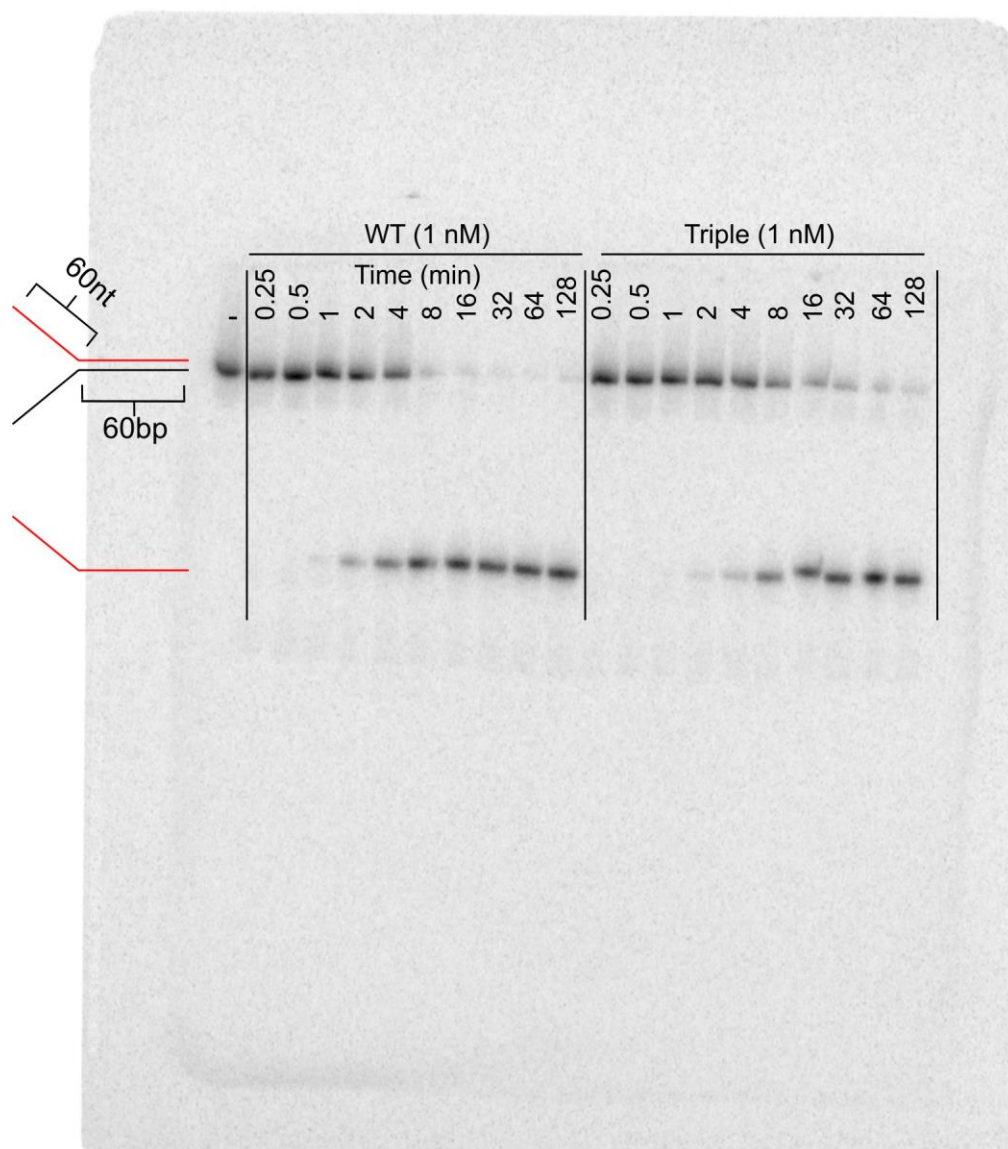

K.

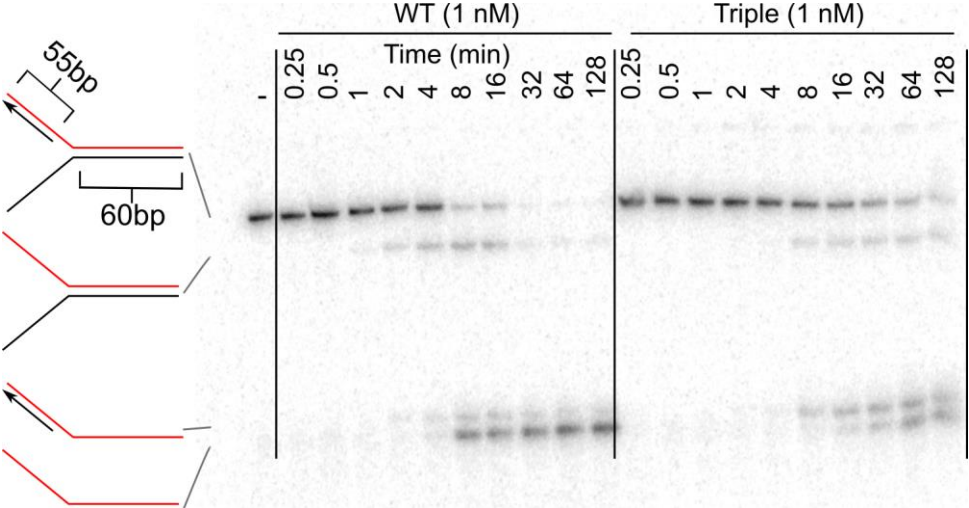

L.

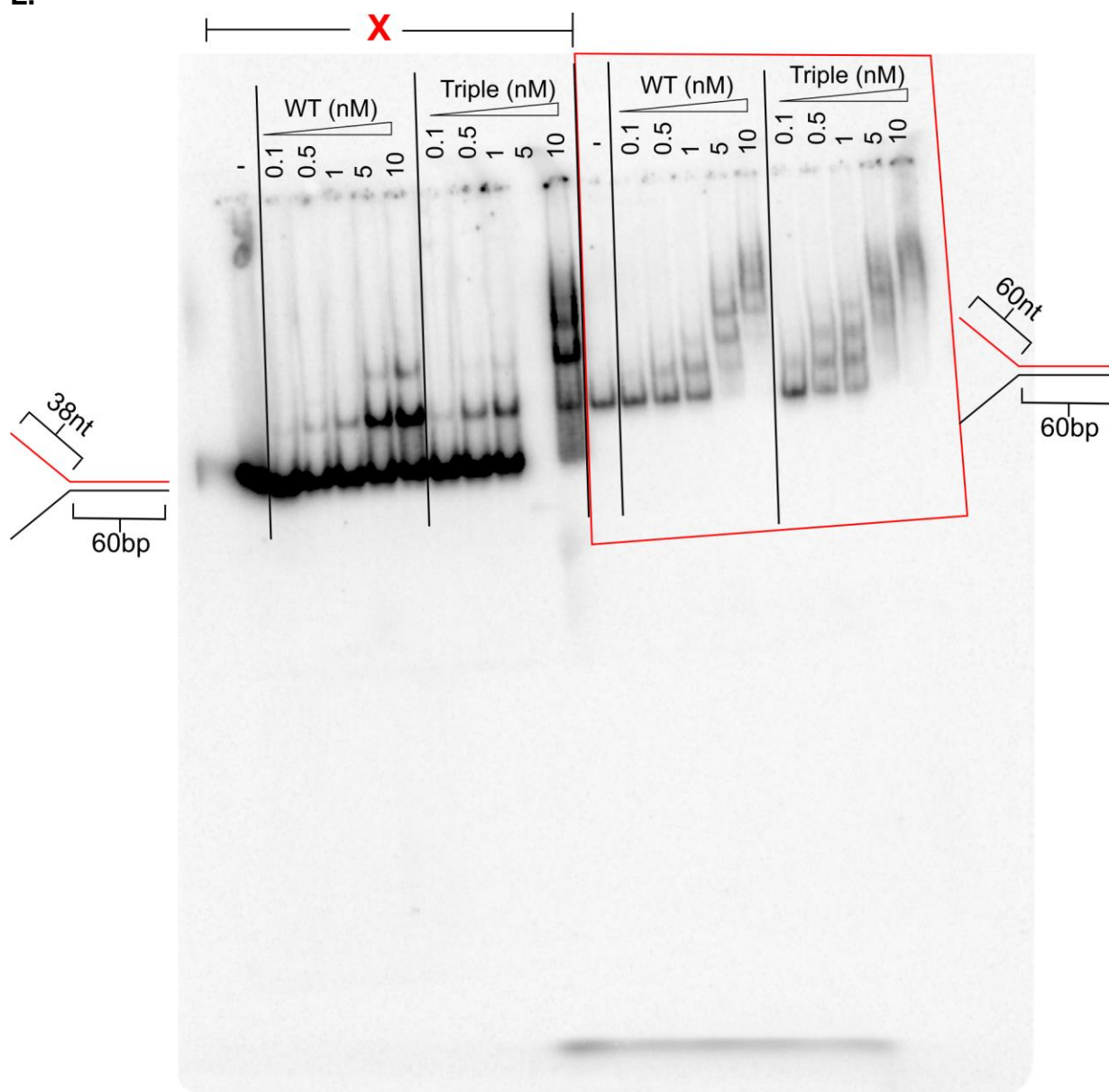

Supplement: S1 Raw images — For gels in which only a portion was used in the respective figure, the portions that were used are boxed. Gel lanes not included in the final figures are marked with a red "X". All images were obtained using a phosphorimager. (A and B) Fig 2A. (C and D) Fig 2B. (E and F) Fig 3A. (G) Fig 3C. (H) Fig 3D. (I) Fig 4A and 4C. (J) Fig 4B. (K) Fig 4D. (L) Fig 5. (PDF) [file pone.0255409.s002.pdf]
