# Supplementary material for: Examination of the roles of a conserved motif in the PriA helicase in structure-specific DNA unwinding and processivity
Source: PLoS One. 2021 Jul 30;16(7):e0255409. doi: 10.1371/journal.pone.0255409 (PMC8323898; doi:10.1371/journal.pone.0255409)
Supplement: S1 Table — (PDF) [file pone.0255409.s003.pdf]

Table S1: Oligonucleotides used to prepare synthetic forks

Name	Sequence (5′→3′)	Source	Fork structure
1b-98: Leading template	GCAAGCCTTCTACAGGTCGACCGTCCATGGCGACTCGAGACCGCAATAC GGATAAGGGCTGAGCACGCCGACGAACATTCACCACGCCAGACCACGTA	(22)	Two-, three-, and four-stranded fork
3L-98: Lagging template	GACTATCTACGTCCGAGGCTCGCGCCGCAGACTCATTTAGCCCTTATCC GTATTGCGGTCTCGAGTCGCCATGGACGGTCGACCTGTAGAAGGCTTGC	(22)	Two-, three-, and four-stranded fork
b-33: Nascent lagging	AGTCTGCGGCGCGAGCCTCGGACGTAGATAGTC	(22)	Three-, and four-stranded fork
11b-38: Nascent leading	TACGTGGTCTGGCGTGGTGAATGTTCGTCGGCGTGCTC	(22)	Four-stranded fork
oTW140: Extended leading template	GCAAGCCTTCTACAGGTCGACCGTCCATGGCGACTCGAGACCGCAATAC GGATAAGGGCTGAGCACGCCGACGAACATTCACCACGCCAGACCACGTA GTGCTATAAGCTTCGCTGACGT	(19)	Extended two-, three-stranded fork
oTW141: Extended lagging template	AGCGTAACGGATCGACCGCTTTGACTATCTACGTCCGAGGCTCGCGCCG CAGACTCATTTAGCCCTTATCCGTATTGCGGTCTCGAGTCGCCATGGACG GTCGACCTGTAGAAGGCTTGC	(19)	Extended two-, three-stranded fork
oTW143: Extended nascent lagging	AGTCTGCGGCGCGAGCCTCGGACGTAGATAGTCAAAGCGGTCGATCCGT TACGCT	(19)	Extended three-stranded fork
